# Heavy metals in sands of sandboxes: health risk associated with their quantities and form of occurrence in some spas of Poland

**DOI:** 10.1007/s11356-017-9531-2

**Published:** 2017-07-06

**Authors:** Alicja Kicińska, Magdalena Mamak, Monika Skrzypek

**Affiliations:** Faculty of Geology, Geophysics and Environmental Protection, AGH University of Science and Technology, Al. Mickiewicza 30, 30-059 Krakow, Poland

**Keywords:** Heavy metals, Sandboxes, Risk assessment, Health resorts, CEE

## Abstract

The authors dealt with some hazardous elements, i.e. As, Cd, Co, Cr, Cu, Ni, Pb and Zn, contained in sands of the sandboxes localized in playgrounds of seven spas in southern Poland (CEE). The following determinations were made: the total contents of metals, the most mobile metals (water-leachable fraction) and the metals available to plants and organisms (CaCl_2_- and EDTA-extractable fractions). The totals of the metals are below the upper limits of the values recommended for soils of the protected areas (type A). The mobility of the metals is low: the forms leachable with water range from 0.7% of the total content (TC) of Pb to 13.4% TC of Cd. The forms available to living organisms contain considerably higher quantities of the metals: from 2.3% TC of Ni to 22.6% TC of As in CaCl_2_-extractable fractions and from 0.7% TC of Cr to 82% TC of As in EDTA-extractable fractions. An assessment of the health risk indicates that children are exposed to the metals present in the sandboxes mostly due to inadvertent swallowing of “dirt” from their hands. The highest are the HQ_ingestion_ indexes of As and Cr, both for the 3-year-old permanent spa residents (5.74E−02 and 1.71E−02, respectively) and the spa visitors of the same age (7.47E−03 and 2.22E−03, respectively) and the 6-year-old residents (4.31E−02 and 1.28E−02, respectively) and visitors (5.60E−03 and 1.66E−03, respectively). The health risk indexes *HI* in the case of non-cancerogenic substances for children 3 and 6 years old are for spa residents, 9.59E−02 and 7.19E−02, respectively, and for children visitors, who are exposed to environmental factors for a much shorter time than the residents, 1.25E−02 and 9.35E−03, respectively. All the risk indexes have their values significantly below 1, which proves the lack of deleterious effects resulting from the exposition of children to the elements considered. The children of both age groups, exposed to the cancerogenic substances, are endangered mainly by As. The risk values of the cancerogenic As for 3- and 6-year-old children residents are 1.27E−06 and 1.90E−06, respectively, and for children visitors of the same ages 1.65E−07 and 2.47E−07, respectively. These values are significantly lower than a permissible level of "1∙10E-05" and means that also in this case, the health risk is minimal. The risk values calculated for the remaining metals are much lower and follow the sequence Cr > Co > Cd. However, an adverse impact of some sand-contained pollutants that are attributed to the motor traffic (Cu, Zn, Ni, Cr, Co and Pb) and low emissions (mainly As and Cd) has been established in the spa resorts in question.

## Introduction

Spas are defined as the localities in which the medical treatment based on natural resources, i.e. mineral and therapeutic waters, peloids and therapeutic gases, is offered. Each spa has its specific medicinal profile and offers bathing, crenotherapy, inhalations, irrigations and rinsing, depending on the infrastructure available (hospitals, sanatoriums, ambulatories or physiotherapeutic facilities).

It is rather difficult to establish the number of spas in the world because of various definitions of the very concept of the “spa” term. An estimation gives a number of over 590 localities fulfilling all the criteria ascribed to the spas proper (health resorts); however, spa-like localities and the localities with substantial resources of thermal waters also pretend to be included into this group (Dryglas and Różycki 2016). The number of such spa-pretending centres in Europe itself is above 200, mainly in Hungary and Slovakia. The leaders of the statutory spa localities in Europe are Germany (268), Austria (96), Poland (45), France (44) and the Czech Republic (40). Outside Europe, the country with the highest number of statutory spas is Japan (76) that is also regarded as the birthplace of therapeutic thermal bathing (Smith and Puczko 2009).

Apart of the therapeutic treatment that uses natural balneological resources, it is the long-lasting stay in spa resorts that matters but only if their climate brings about non-questionable medical advantages. The duration of such periods is usually 2–3 weeks, and a stay should be spent in a microclimate free of pollutants. Only then will result the spa staying in a detoxication of human organisms and a general health improvement.

Legislation systems of major European countries contain stipulations enforcing local administrations to pursue a proper environmental policy, based not only on the sustainable management of natural balneological resources, but also on a taking care of the whole infrastructure, including water and sewerage systems, energy sector, public transport and waste management. Considering such a policy that is aimed at providing and maintaining a proper microclimate within spas, the resort localities are usually divided into internal protection zones. For instance, in Poland, there are three zones distinguished (A, B and C), two with a specified contribution of green areas: zone A—75%, zone B at least 55%, while zone C plays a role of a buffer to zone B; this division should provide a comfort of stay and a proper recuperation of spa visitors (Minister 2005). The spa green areas include first of all not only parks, but also promenades, playgrounds and recreational facilities. These are just the places where the spa visitors spend most of their time in the open. The role of playgrounds is specific. Equipped in sandboxes, toys and other playing facilities, they attract children, who spend there at least several hours a day during their stay in the spa. Unfortunately, despite an overall public awareness and preventive measures, spas are also exposed to pollution, mainly associated with the motor traffic: for instance, a considerable majority of spa visitors use their private cars. Heating houses with coal (usually of inferior quality, sometimes with coal muds), fine coke and/or wood is another source of pollution in Polish spas, particularly those in the mountainous parts of the country. This problem, called the low emission, becomes serious mainly in the heating season (late autumn–early spring), when the number of visitors is also high and their living quarters must attain adequate temperatures (Siudek et al. 2016). In many spas, there is no regular environmental monitoring; therefore, the question “whether a longer stay in the spa is healthy for its visitors or not” is fully justified. It is particularly important in the case of youngsters, because their being exposed to the presence of hazardous elements or substances in elevated doses may cause negative health effects (Szyczewski et al. 2009; Siudek et al. 2015). Into this dangerous group definitely belong heavy metals, particularly Cd, Co, Cr, Pb and As (the latter formally a metalloid), which not only disturb proper functioning of young organisms, but may also irreversibly affect their further, proper development (Kabata-Pendias et al. 1993).

The health of the humans exposed to heavy metals is endangered considering three access paths of pollutants: they enter an organism by breathing, swallowing (common at children who often put dirty hands into their mouths) and skin penetrating. In all three cases, potential toxic elements are contained mainly in the finest fractions of airborne dusts (PM_10_, PM_2.5_), whose quantities of various organic and inorganic compounds are not always neutral to humans (Kicińska 2016a, b; Pysz et al. 2016; Zwozdziak et al. 2016).

Heavy metals regardless of their origin (natural or anthropogenic) represent a serious environmental threat, resulting mainly from their relatively easy bioaccumulation and biomagnification. Metabolism of some of them (e.g. Cu, Cd and Zn) is controlled by genetic factors and is associated with the capability of organisms to synthesize metaloenzymes and metaloproteins (for instance, metalothionine), which increase resistance to toxic effects of metals (Erway 1984; Ernst 1996). According to Szpunar et al. (2003), human organisms can complexate trace elements predominantly by proteins and their constituents (via the sulphur atom in the case of Cu, Zn and Cd and the oxygen atom in the case of Co, Ni, Cu and Zn), nucleic acids and their constituents (Cr, Ni), polysaccharides (Pb), tetrapyrrole ligands (Co and Ni) and small organic ligands (Ni). Some of the metals mentioned are indispensable for a proper functioning of the organism. For instance, Co is contained in cobalamine (vitamin B_12_) and plays an essential role in the formation of red blood cells and the metabolism of nucleic acids and proteins. Chromium is contained in trypsin and is an important factor of metabolism of glucose, some proteins and fats. A deficiency of Zn disturbs the development of the bone system and the reproductive functions and affects skin causing its inflammation. On the other hand, a long-lasting excess not only of Zn, but also of As and Ni, in the organism may be carcinogenic. Excessive quantities of Cu and Pb in children’s organisms result in disorders of the brain and physical development and are a cause of liver diseases.

Investigations on the health risk of children arising from the presence of metals in the environment were carried out so far mainly in playgrounds and urban parks of larger agglomerations with industrial facilities (De Miguel et al. 2007; Figueiredo et al. 2011; Namik et al. 2012; Verla et al. 2015). The subjects of these studies included soils (Diatta and Grzebisz 2011; Figueiredo et al. 2011; Guney et al. 2010; Stajic et al. 2016; Verla et al. 2015), urban dusts (Boldo et al. 2006; Kicińska and Bożęcki 2017; Khaniabadi et al. 2016; Lim et al. 2011; Mazzei et al. 2008; Yu et al. 2013; Wang et al. 2016; Zhang et al. 2015) and road dusts (Du et al. 2013; Ferreira-Baptista and De Miguel 2005). Only subordinate are the papers devoted to pollution of health resorts and their playing grounds, not saying on sandboxes and the sand that fills them (Jasiewicz et al. 2009; De Miguel et al. 2007; Nieć et al. 2013), although these facilities are most willingly used by children playing outdoor.

Considering the problems described, in seven spas of southern Poland (CEE), the sand of playground sandboxes was sampled in 2016 and the analyses, followed by recalculations and evaluations of their results, included the following:

1. The total contents (TC) of selected heavy metals associated with the motor traffic (Cu, Zn, Ni, Cr, Co and Pb) and the low emissions (mainly As and Cd)
2. The contents of easily soluble metals, i.e. those more mobile and bioavailable (e.g. for plants and soil organisms)
3. An identification of the source(s) of the metals
4. Calculations of the hazard quotient (HQ_*i*_) of three access routes of the elements that enter the human organism (HQ_ingestion_, HQ_inhalation_, HQ_dermal_) and the hazard index (*HI*) for threshold-acting (non-cancerogenic) substances and cancer risk for non-threshold-acting (cancerogenic) substances in the case of children frequenting the playgrounds and being exposed to the toxic elements. Two groups of children have been distinguished in the investigations: those being the permanent inhabitants of the spa (i.e. residents) and those coming there only for shorter stays (i.e. visitors).

## Sampling sites

**Fig. 1:**
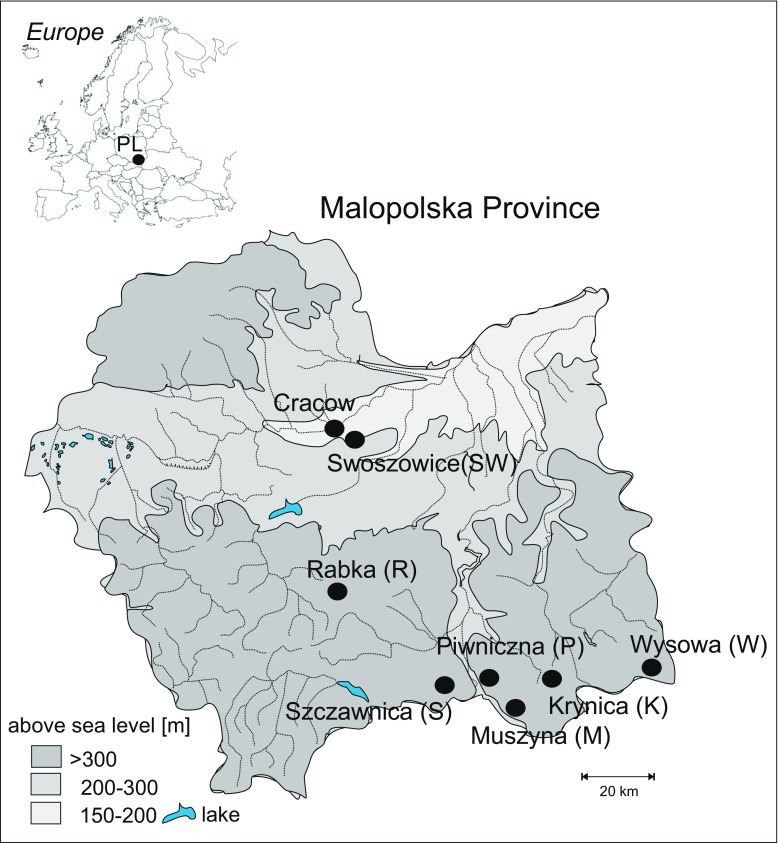
Location of sampling sites withing the border of Malopolska Province

The spas in question are located in the Polish part of the Carpathian Mountains, in the Małopolska Voivodeship (Fig. 1), a region located within the moderate climate with transitional features. The spa status was granted all the seven localities in the same time (1967). Six of them, i.e. Krynica, Muszyna, Rabka, Piwniczna, Szczawnica and Wysowa, are situated in foothill and mountainous bioclimatic regions, where the climate conditions strongly vary and are highly stimulating. The seventh of them, Swoszowice, is located in the lowermost, northern margin part of the Carpathian Foothills, and represents a subregion of an elevated thermal stimulation. Mineral waters are the major medicinal natural assets of all the spas, and the spectrum of therapeutic treatments ranges from four to ten (Table 1). For instance, the disorders of the upper and lower respiratory tracts are treated in the six aforementioned spas, with Swoszowice being again an exception.

**Table 1:** Sampling sites and short characteristic of spas

Health resort (symbol)	Sampling site	Coordinance		Characteristics of the playground area	Number and kinds of medicinal treatment^a^	Natural healing resources/the healing properties of the climate
		E	N			
Szczawnica (S)	S1	20° 28′ 09.69″	49° 25′ 36.59″	Private area, next to main road	5: A, C, F, G, I	Mineral water/mountain climate, moderately incentives, moderated by forest areas (stands of conifers)
	S2	20° 28′ 58.24″	49° 25′ 31.41″	Solar sanatorium, next to way exit		
	S3	20° 29′ 09.37″	49° 25′ 28.94″	Nursery area		
Rabka (R)	R1	19° 57′ 44.37″	49° 36′ 19.41″	In urban park	6: A, D, F, G, I, M	Mineral water/mountain climate, moderately and strongly incentives
	R2	19° 57′ 24.36″	49° 36′ 37.60″	Next to nursey		
	R3	19° 57′ 16.74″	49° 36′ 37.06″	Next to block of flats		
Krynica (K)	K1	20° 57′ 16.70″	49° 25′ 41.81″	Next to block of flats	8: B, C, F, H, I, N, O, P	Mineral water/subalpine climate, moderated by forest areas
	K2	20° 55′ 50.80″	49° 24′ 37.39″	On hotel area		
	K3	20° 56′ 45.53″	49° 24 01.57″	Next to block of flats		
Muszyna (M)	M1	20° 53′ 38.13″	49° 21′ 13.57″	Private area, next to mail road	4: C, F, H, K	Mineral water/piedmont climate, moderately and strongly incentives, moderated by forested mountain slopes
	M2	20° 53′ 16.87″	49° 21′ 00.59″	Recreation area, next to rope park		
	M3	20° 53′ 34.57″	49° 21′ 06.21″	Private area, next to mail road		
	M4	20° 53′ 39.59″	49° 21′ 05.07″	Next to block of flats		
Piwniczna (P)	P1	20° 43′ 03.72″	49° 26′ 25.19″	In urban park, next to river	4: C, F, G, H	Mineral water/mountain climate, moderately and strongly incentives
	P2	20° 42′ 37.94″	49° 26′ 29.64″	Next to block of flats		
	P3	20° 42′ 41.77″	49° 26′ 28.81″	Next to block of flats		
	P4	20° 42′ 50.88″	49° 26′ 23.84″	Private area, next to mail road		
Wysowa (W)	W1	21° 10′ 57.02″	49° 26′ 24.74″	In urban park	10: A, B, C, F, G, H, I, J, L, O	Mineral water/mountain climate, gentle incentives, moderated by forest areas (beech, spruce, fir)
	W2	21° 10′ 49.05″	49° 26′ 20.85″	In urban park		
	W3	21° 10′ 20.64″	49° 26′ 20.14″	Next to block of flats		
Swoszowice (SW)	SW1	19° 55′ 55.88″	49° 59′ 32.55″	Next to block of flats	5: A, B, C, L, M	Mineral water/lowland climate, moderately incentives, microclimate zone
	SW2	19° 56′ 43.16″	49° 59′ 39.94″	Next to nursey		

^a^Treatment of diseases: *A* orthopaedic trauma, *B* nervous system, *C* rheumatologic, *D* cardiology and hypertension, *E* peripheral vascular, *F* upper respiratory tract, *G* lower respiratory tract, *H* digestive system, *I* diabetes, *J* obesity, *K* endocrine, *L* osteoporosis, *M* skin, *N* feminine, *O* kidney and urinary tract, *P* blood and cardiovascular system, *R* eye and appendages of an eye

In each of the seven spas were identified from two to four playgrounds with sandboxes. The sand samples were collected in four corners of each sandbox, each of the samples weighing around 0.5 kg; a total of 88 samples represented thus the initial study set. Next, the four samples from every sandbox were mixed together and homogenized; in this way, 22 general samples with the weight around 2 kg each were obtained. The field campaign covered the period of September and October 2016.

### Laboratory methods

The study material was dried, homogenized once again and prepared for chemical analyses. They were carried out at the Laboratory of Trace Analyses at the Department of the Environment Protection, AGH University of Science and Technology. In the first stage, the grain fraction >2 mm was removed and the remaining <2-mm analytical samples had their pH determined in water according to the standard PN-ISO 10390. The most mobile metals were assayed after their leaching with distilled water at the solid to water ratio 1:10.

The determinations of the bioavailable (e.g. for plants and soil organism) quantities of metals were conducted after a 2.5-h extraction of the analytical samples with 0.01 M solution of CaCl_2_ at the solid-to=liquid ratio 1:10 (Rauret 1998; Pueyo et al. 2004) and with 0.02 M solution of EDTA at the solid-to-liquid ratio 1:10. EDTA is used extensively in soil sciences to determine the bioavailability of trace elements (including metals) and possible decontamination methods of polluted soils. Since the EDTA leaching is of a non-selective nature, the co-dissolution of major elements also takes place (Manouchehri et al. 2006; Lo and Yang 1999). In the case of the CaCl_2_ extraction, the 0.01 M solution of calcium chloride has approximately the same ionic strength as an average salt concentration in many soil solutions (Houba et al. 2000).

The grain fractions <2 mm were dissolved in a mineralizer at the temperature 130 °C in the 3:1 mixture of concentrated HCl and HNO_3_ to establish total contents (TC) of elements. Their concentrations in the solutions were determined at the certified Hydrogeochemical Laboratory at the AGH University of Science and Technology using the ICP-MS method (an Elan 6100 apparatus). The certificate No. AB1050 has been issued in Poland by the State Accreditation Commission (Polish abbreviation PCA). The Certipur Certified Reference Material (HC69208280) was used as a standard. The analysis precision for As, Cd, Co, Cr, Cu, Ni, Pb and Zn was 10% and the accuracy 95–104%. The limits of detection (LOD) and limits of quantification (LOQ) were calculated from the following equations:

1 $$ \mathrm{LOD}={X}_b+3{SD}_b $$

2 $$ \mathrm{LOQ}={X}_b+10{SD}_b $$

where

Xb: the mean concentration of the blank (zero concentration) sample and
*SD*_b_: the standard deviation of the blank

and their values are presented in Table 2.

**Table 2:** Total concentration of metals and metalloid in sand material (fraction <2 mm)

Sampling site parameter		As	Cd	Co	Cr	Cu	Ni	Pb	Zn
		(mg/dm^3^)							
CRM	Certified value	98 ± 5	9.9 ± 0.5	9.9 ± 0.5	9.9 ± 0.5	9.9 ± 0.5	9.9 ± 0.5	9.8 ± 0.5	99 ± 5
	Measured value (TA)	92.88 (94.8)	9.53 (96.3)	9.82 (99.2)	10.16 (102.7)	9.64 (97.2)	10.16 (103.7)	10.07 (101.7)	102.6 (103.6)
LOD		0.0358	0.0013	0.0008	0.0039	0.0015	0.0064	0.0323	0.0473
LOQ		0.1552	0.0055	0.0036	0.0139	0.0064	0.0216	0.1198	0.2053
Szczawnica (mg/kg)									
Min.–max.		1.05–1.94	0.036–0.039	0.67–0.81	1.04–4.35	1.52–181	2.71–3.47	0.75–2.29	8.13–15.96
Av.		1.50	0.04	0.74	4.19	1.67	3.09	1.52	12.04
Rabka									
Min.–max.		1.22–3.09	0.05–0.13	0.21–0.84	2.40–4.43	1.04–1.64	1.46–3.67	1.44–1.81	10.07–13.95
Av.		2.15	0.09	0.52	3.41	1.34	2.56	1.63	12.01
Krynica									
Min.–max.		1.35–1.62	0.05–0.18	0.49–1.39	3.28–5.13	1.15–2.01	2.04–5.08	1.25–4.31	10.78–18.67
Av.		1.48	0.11	0.94	4.21	1.58	3.56	2.78	14.72
Muszyna									
Min.–max.		1.11–1.37	0.01–0.03	0.90–1.09	4.68–5.48	1.43–1.75	3.56–4.22	1.05–1.53	7.59–9.68
Av.		1.24	0.02	1.00	5.08	1.59	3.89	1.29	8.63
Piwniczna									
Min.–max.		0.20–0.52	0.01–0.03	0.79–1.53	4.78–5.43	2.18–2.24	3.56–5.37	0.68–2.37	9.51–10.19
Av.		0.36	0.02	1.16	5.11	2.21	4.47	1.52	9.85
Wysowa									
Min.–max.		0.77–1.05	0.01–0.06	0.73–1.59	4.26–6.02	1.45–1.98	3.30–5.07	0.86–2.35	7.71–13.58
Av.		0.91	0.04	1.16	5.14	1.72	4.19	1.60	10.65
Swoszowice									
Min.–max.		1.52–3.60	0.11–0.13	0.57–0.61	3.25–3.29	0.56–0.73	2.29–2.37	2.52–5.50	15.20–37.92
Av.		2.56	0.12	0.59	3.27	0.65	2.33	4.01	26.56
For all destination									
Min.–max.		0.20–3.60	0.01–0.18	0.21–1.59	2.40–6.02	0.56–2.24	1.46–5.37	0.68–5.50	7.59–37.92
Av. ± SD		1.46 ± 0.89	0.06 ± 0.05	0.87 ± 0.39	4.34 ± 0.98	1.54 ± 0.50	3.44 ± 1.16	2.05 ± 1.33	13.50 ± 7.51
Upper limit (% of samples above limit)	A^a^	20 (0%)	1 (0%)	20 (0%)	50 (0%)	30 (0%)	35 (0%)	50 (0%)	100 (0%)
	B^a^	20 (0%)	4 (0%)	20 (0%)	150 (0%)	150 (0%)	100 (0%)	100 (0%)	300 (0%)
	DT^b^	29 (0%)	0.8 (0%)	9 (0%)	100 (0%)	36 (0%)	35 (0%)	85 (0%)	140 (0%)
	NC^c^	20 (0%)	10 (0%)	–	5^d^ (36%)	–	135 (0%)	100 (0%)	-

*CRM* Certified Ref. Material, *AO* analysis trueness (%), *LOD* limits of detection, *LOQ* limits of quantification, *av.* arithmetic average, *SD* standard deviation

^a^According to Minister (2002) *A* protecting areas, *B* other than protecting and industry areas

^b^ *DT* Dutch Target and Intervention values for earth/sediment (Ministerie…2000, www.esdat.net2000)

^c^ *NC* Norway quality criteria for soil day-care centres, playgrounds and schools (Alexander 2006)

^d^For Cr^6+^

The model used in this study to calculated children’s exposure to metals in health resorts was adapted according to the US Environmental Protection Agency (US EPA 1996, 1997). The health risk HQ_*i*_ was calculated accepting three pathways: HQ_ingestion_—direct ingestion of substrate particles, HQ_inhalation_—inhalation of re-suspended particles via mouth and nose and HQ_dermal_—dermal absorption of metals from the particles adhered to an exposed skin (De Miguel et al. 2007). The health risk values: HQ_ing_, HQ_inhal_ and HQ_dermal_ were calculated for each element as the ratio of the contact dose (*D*_ing_, *D*_inhal_ and *D*_dermal_, respectively) divided by the corresponding reference doses (RfD_ing_, RfD_inhal_ and RfD_dermal_, respectively) after US EPA (2005).

The dose was calculated using the following equations (US EPA 1986):

3 $$ {D}_{\mathrm{ingestion}}= C\cdot \frac{\ \mathrm{IngR}\kern0.5em \cdot \mathrm{EF}\kern0.5em \cdot \mathrm{ED}}{\mathrm{BW}\kern0.5em \cdot \mathrm{AT}}\cdot \mathrm{CF}1 $$

4 $$ {D}_{\mathrm{inhalation}}= C\cdot \frac{\ \mathrm{InhR}\kern0.5em \cdot \mathrm{EF}\kern0.5em \cdot \mathrm{ED}}{\mathrm{PEF}\cdot \mathrm{BW}\kern0.5em \cdot \mathrm{AT}} $$

5 $$ {D}_{\mathrm{dermal}}= C\cdot \frac{\mathrm{SA}\cdot \mathrm{SL}\cdot \mathrm{ABS}\cdot \mathrm{EF}\cdot \mathrm{ED}}{\mathrm{BW}\cdot \mathrm{AT}}\cdot \mathrm{CF}1 $$

where

*C*: mean heavy metal concentration in a sample (mg/kg);
IngR: conservative estimates of dust ingestion rates; for children 100 (mg per h) (US EPA 2011);
InhR: inhalation rate; in this study, 1.26 (m^3^/h, for 3 to <6 years old, for moderate intensity) (US EPA 2011);
EF: exposure frequency; for residents accepted as 646 (h/year) after De Miguel et al. (2007), for visitors as 84 (h/year) accepting that most of children stay in the spa for 3 weeks and use playgrounds for 4 h per day; many of them spend every year holidays in the same child health resort;
ED: exposure duration; for younger children 3 (years) and older ones 6 (years);
BW: body mass; for children 3 years old 15 (kg), for those 6 years old 20 (kg) (mean values, US EPA 2011);
AT: averaging time. In the case of non-carcinogens: for 3 year-old children, its value is 3 × 365 = 1095 (days), for 6-year-old children, its value is 6 × 365 = 2190 (days), while in the case of carcinogens, its value is 70 × 365 = 25,550 (days);
SA: exposed skin area, 2800 (cm^2^) (recommended values for mean solid adherence to skin (US EPA 2011)
SL: skin adherence factor, 0.07 (mg/cm^2^ per h) (after De Miguel et al. 2007);
ABS: dermal absorption factor, 0.001 (all the elements except As, for As 0.03) (after De Miguel et al. 2007);
PEF: particle emission factor, 6.8E+08 (m^3^/kg) (after De Miguel et al. 2007);
CF1: unit conversion factor of 10^−6^.

The total hazard index (*HI*) was calculated as a sum of HQ_ing_, HQ_inhal_ and HQ_dermal_ values*.* It is accepted that at the *HI* ≤ 1 adverse health effects are of a low probability to occur, at the *HI >* 1, negative health effects are probable, while the *HI* > 10 values are a sign of a high exposure and a highly possible chronic health risk caused by a toxic factor(s).

For carcinogens, their doses were multiplied by the respective slope factors (SF) to produce a level of the cancer risk. The toxicity values used in the analysis were taken from US EPA (2005). For Pb, the reference doses are those contained in the World Health Organization’s Guidelines for Drinking Water Quality (WHO 1993). The toxicity values of dermal absorption were taken from IRIS (US EPA 2005): the oral reference doses are multiplied while the slope factors divided by a gastrointestinal absorption factor to yield the corresponding dermal values.

The stations that measure the amounts of PM_10_, PM_2.5_ and the metals that the samples contain are only occasionally situated in the parks of the spa resorts of Poland. In the case presented, neither of the parks considered in the report has such a station. An interpolation to the park areas of the data obtained in other sites of the spa (open markets, streets, major thoroughfares, etc.) would have been burdened with much higher errors than our calculation of a risk attributed to inhaling airborne particles. This is a way to estimate risk from inhalation; the older method (US EPA 2005), based on IR and BW, was chosen.

Statistical calculations and data presentations were conducted with the Statistica ver. 10 and Excel applications. The group analyses were based on variables (concentrations of elements) characterizing the objects considered (i.e. parks) and allowed distinguishing the groups (clusters), inside which the parameters selected are more similar within the given group than to the parameters within other groups. This type of analysis allows to establishing whether the groups reveal any regularity (correlation), and as a result, it reduces the databases to the averages calculated for specific groups. The Ward method is an analysis of a variance problem, based on minimizing totals of square deviations within the groups (clusters). The method accumulates into clusters the cases with minimum diversifications. The differences between means were detected by Turkey’s test at a significance level of 0.05.

## Results of investigations

### Mineral and chemical composition of sands

**Fig. 2:**
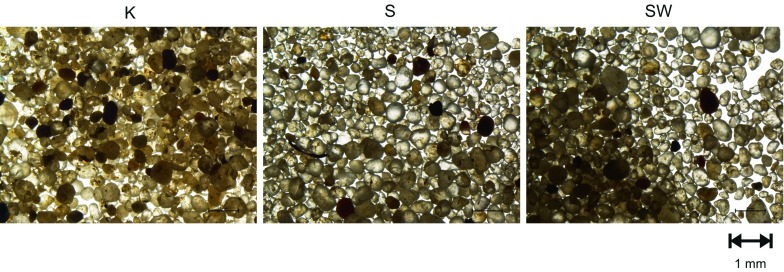
Images of the test material seen under an optical microscope. *K* sample please change "form" to "from" - 3 times Krynica, *S* sample from Szczawnica, *SW* sample from Swoszowice

Each of the general samples is mostly, in around 90%, composed of oval, well-rounded quartz grains. Their sizes measured under an optical Nikon Eclipse 50i-POL microscope range between 0.1 and 0.5 mm, with a distinct prevalence of the grain fraction 0.3–0.5 mm (Fig. 2). The quartz grains are highly transparent and colourless or with a delicate milky brown tint. Opaque grains make up 7–8% and represent probably amphiboles and pyroxenes. Greenish grains of olivines occur occasionally. The list is supplemented by micas, occurring as fine, disintegrating flakes of both muscovite and biotite.

The As contents range from 0.2 to 3.6 mg/kg (Table 2) and those of Cd from 0.01 to 0.18 mg/kg. The contents of Co, Cr, Cu and Ni are comparable: 0.21–1.59, 2.40–6.02, 0.56–2.24 and 1.46–5.37 mg/kg, respectively. Zinc occurs at distinctly higher quantities, from 7.59 to 38 mg/kg.

The highest TC values of As, Cd, Pb and Zn occur in Swoszowice (Fig. 1), while those of Co, Cu and Ni (1.16, 2.21 and 4.47 mg/kg, respectively) in Piwniczna. The highest TC of Cr (range 4.26–6.02, mean 5.14 mg/kg) was determined in Wysowa. The distribution of the lowest metal quantities is as follows: of Cd, Pb and Zn in Muszyna, of Co in Rabka, while of Cr, Cu and Ni in Swoszowice.

Owing to the lack of standards pertaining to the content of heavy metals in sands of the playground sandboxes, the results were compared with the metal standards for soils and sediments effective in several European countries. In the Polish legislation (Minister 2002), three types of areas have been distinguished: protected (group A), industrial (group C) and others than protected and industrial (group B). All the results obtained for the sands of seven Polish spas do not exceed the permissible values of the group A soils, i.e. As 20, Cd 1, Co 20, Cu 30, Pb 50, Zn 100, Cr 50 and Ni 35 mg/kg. The results are also lower than the respective values of the B group soils.

Different permissible contents of heavy metals in grounds, soils and other sediments can be found in almost all environmental regulations of various countries. Distinctly more restrictive are Dutch standards (Ministerie 2000): their Cd and Co permissible limits are much lower, while those of Cr, Pb and Zn slightly lower than the Polish equivalents of the group A soils; the only comparable are the permissible values of As, Cu and Ni (Table 2). Thus, none of the sand samples of the spas exceeds the As, Cr, Cd, Pb, Ni, Co, Zn and Cu limits of the Dutch Target (Ministerie…^2000^). The Norwegian standards of the soils occurring in playgrounds and in the green areas around schools (Alexander 2006) are distinctly less restrictive than the Polish ones in the case of Cd, Ni and Pb contents, but are much higher in the case of Cr. No permissible values of Co, Cu and Zn have been set in Norway. Classifying our samples according to the Norwegian requirements, the contents of As, Cd, Pb and Ni are below the upper limits, but 36% samples exceed the stipulated upper content of 5 mg/kg of Cr.

The sand samples may also be assessed using the guidelines of the IUNG-PIB (Institute of Soil Science and Plant Cultivation) in Puławy, Poland (Kabata-Pendias et al. 1993). The IUNG classifies the soils into six groups on the basis of their contamination, from class 0—the quantity of an element corresponds to its natural content—to class VI—soils of a very high degree of pollution. According to this classification, the samples of sands belong to the category of materials non-polluted with Pb, Zn, Cu, Ni and Cd, which means that the contents of these elements fall into the range of natural values. The limits of the remaining metals, i.e. As, Co and Cr, have not been specified in the IUNG guidelines.

### Sources of heavy metals in sandboxes

**Fig. 3:**
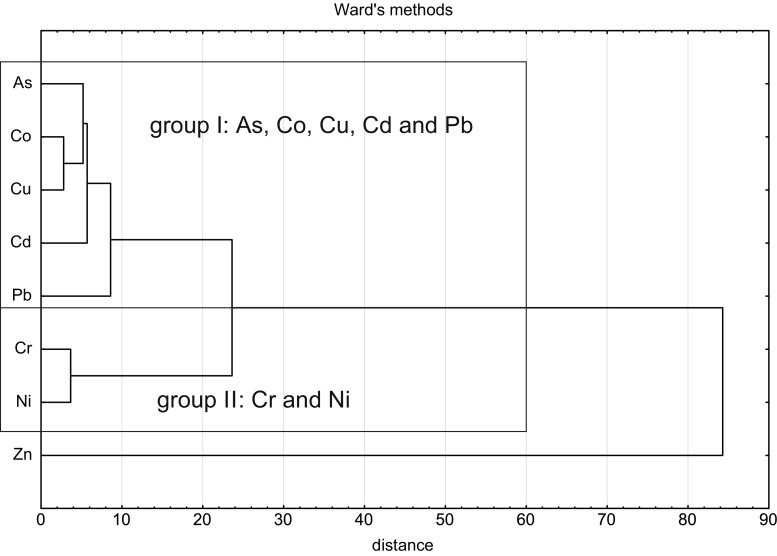
Dendrogram for specified element content in sand samples from spas

The metals included into the group of harmful elements may usually be ascribed into several subgroups, each of them of the common genesis, i.e. generated by the same emitter type. The sources of the metals present in the sands have been established by calculating correlation coefficients of these elements (Table 3) and conducting the cluster analysis (Fig. 3).

**Table 3:** Correlation coefficients (*r*_*xy*_) calculated for the concentration of metals and metalloid in sands material (fraction <2 mm)

Element	Cd	Co	Cr	Cu	Ni	Pb	Zn
As	0.60***	−0.27*	−0.35***	−0.59	0.72***	0.15	0.12***
Cd	–	−0.53*	−0.60***	−0.64***	−0.57***	0.62***	0.53***
Co		–	0.93***	0.72***	0.98***	−0.23**	−0.33***
Cr			–	0.82***	0.95*	0.89***	−0.47***
Cu				–	0.80***	−0.51	−0.55***
Ni					–	−0.38	−0.45***
Pb						–	0.91***

Bold values high correlation (0.5 ≤ *r* < 0.7), italic bold values indicate very high correlation (0.7 ≤ *r* < 0.9), and italic values indicate the correlation almost full (0.9 ≤ *r* < 1)

Differences between the content, statistically significant at *p ≤ 0.05; **p ≤ 0.01; ***p ≤ 0.001

The correlation coefficients (*r*) point to a high dependence in the range 0.5 ≤ *r* < 0.7 of three pairs: Cd-As, Cd-Pb and Cd-Zn and a very high correlation of 0.7 ≤ *r* < 0.9 of five pairs: Cu-Co, Cu-Cr, Cu-Ni, As-Ni and Cr-Pb. Almost fully correlated (0.9 ≤ *r* < 1) are Co-Cr, Co-Ni, Cr-Ni and Pb-Zn.

The dendrogram (Fig. 3) indicates two clusters, called further groups I and II. Into the group I belong As, Co, Cu, Cd and Pb and into the group II two elements, Cr and Ni. The group I is composed of the elements associated mainly with the low emissions and combustion of motor fuels. Elevated quantities of As, Cd and Co occur in biolites (fossil fuels) and, according to Kabata-Pendias et al. (1993), amount to 5–15, 0.7–1.3 and 5–40 mg/kg, respectively. In the course of burning, mainly in households and small boiler houses, these elements find the way to the atmosphere from which are deposited on the ground surface. Another group of metals includes elements used for production of vehicles, mainly in manufacturing of protective coats. More important and being in a common use are nickel-chromium coats with anti-corrosive properties. Relatively elastic, they show good adhesion to steel or copper parts. Since quickly becoming matt not only when used on the road but due to the normal atmospheric exposure, they are covered by an additional, lustre-providing chromium layer. Also all moving motor parts due to their standard wear are sources of a contamination in the form of fine metal-bearing particles that accumulate in various components of the environment. The most obvious are such wear products of brake discs and shoes, manufactured—among others—with the use of copper and steel fibres and graphite, and also of vehicle tires, not only rubber itself but also tyre metallic mesh reinforcements or wheel rings.

### Sandboxes as a possible source of metals polluting the environment

Two environmental parameters of heavy metals contained in the sand, their mobility and availability to soil organisms and to plants, were determined with the use of extraction methods (Table 4). Water extracts a part of metals weakly bound to solid particles, mainly the metals at the exchangeable positions. Metals in the form of ions are highly mobile in rainfall and surface waters; moreover, 100% of them can penetrate cellular membranes. The extraction with the 0.01 M CaCl_2_ and 0.02 M EDTA releases the metals that are mostly available to living organisms. The extractions with the not buffered solution of CaCl_2_ and with solutions of complexing compounds (EDTA) were applied to determine hazards to the water-soil environment, mainly to surface waters, soil-living species and plants, caused by the metals present in playgrounds (Karczewska et al. 2009).

**Table 4:** Extraction of EDTA and 0.01 M CaC1_2_ solution and water leaching of metal (as a % of total content from sand material) (fraction <2 mm)

Sampling site/parameter			As	Cd	Co	Cr	Cu	Ni	Pb	Zn
			(% of TC)							
Szczawnica										
Water leaching/min.–max.			0.01–0.20	0.2–30.2	0.5–5.6	4.7–5.4	6.1–8.2	2.8–4.0	0.01–0.3	4.5–5.0
0.01 M CaCl_2_ extraction/min.–max.			0.19–0.21	8.7–31.0	0.1–5.5	0.3–5.7	0.4–8.4	0.2–4.4	13.0–25.9	0.7–5.9
EDTA extraction/min.–max.			58–100	41–90	4–10	0.4–0.5	7–20	3–5	13–26	6–13
Rabka										
Water leaching/min.–max.			0.01–0.01	0.2–2.4	2.9–13.4	2.4–4.1	4.4–12.1	1.2–1.4	0.9–3.4	5.2–11.8
0.01 M CaCl_2_ extraction/min.–max.			0.08–0.11	4.5–16.9	1.2–15.9	0.02–4.2	0.7–12.7	0.3–1.9	29.9–78.4	0.6–12.4
EDTA extraction/min.–max.			40–99	36–43	7–29	1–2	8–19	4–9	30–78	21–31
Krynica										
Water leaching/min.–max.			5.1–5.5	0.1–7.3	3.1–3.3	1.7–3.4	6.5–7.0	1.0–2.3	0.1–0.2	1.3–3.7
0.01 M CaCl_2_ extraction/min.–max.			5.1–5.1	1.9–7.1	0.2–3.4	0.2–3.3	0.1–7.5	0.2–2.3	7.4–22.4	0.6–3.9
EDTA extraction/min.–max.			69–89	15–73	6–7	0.6–0.7	20–23	2–4	7–22	7–8
Muszyna										
Water leaching/min.–max.			0.01–3.8	1.0–8.6	4.4–4.5	3.7–4.1	5.0–7.8	2.9–3.1	0.01–0.02	5.7–6.2
0.01 M CaCl_2_ extraction/min.–max.			0.21–5.2	10.3–24.7	0.3–4.5	0.2–4.3	0.7–7.7	0.3–3.5	4.1–7.7	1.4–7.59
EDTA extraction/min.–max.			84–100	46–100	2–3	0.2–0.5	7–10	2.2–2.5	4–10	8–9
Piwniczna										
Water leaching/min.–max.			0.01–76.7	13.3–54.7	2.1–2.8	1.2–2.3	1.9–3.2	0.7–1.5	0.01–0.02	0.4–0.7
0.01 M CaCl_2_ extraction/min.–max.			0.72–81.2	17.5–45. 9	0.3–3.4	0.1–2.2	0.5–3.5	0.3–1.7	0.3–15.8	0.6–1.4
EDTA extraction/min.–max.			100	69–100	3–5	0.5–0.6	5–11	2.4–2.6	13–16	8–14
Wysowa										
Water leaching/min.–max.			0.01–1.9	8.4–60.0	2.2–5.0	3.2–4.0	5.6–5.8	2.3–2.6	0.01–0.03	0.8–1.4
0.01 M CaCl_2_ extraction/min.–max.			0.15–2.4	0.4–62. 9	0.6–5.8	0.1–4.2	0.70–6.8	0.1–2.4	1.5–2.1	0.5–0.5
EDTA extraction/min.–max.			100	44–100	4–7	0.2–0.5	8–9	2.7–2.9	4–14	6–7
Swoszowice										
Water leaching/min.–max.			0.01–0.02	0.2–1.5	6.0–6.5	2.0–3.9	11.3–11.9	1.5–3.2	1.7–3.8	3.5–7.3
0.01 M CaCl_2_ extraction/min.–max.			0.08–0.16	0.8–1.9	0.2–7.8	0.1–4.1	2.1–12.2	0.1–4.1	0.1–4.3	0.7–7.4
EDTA extraction/min.–max.			28–78	33–45	9–16	0.7–0.8	33–38	4–8	23–32	12–14
For all destination										
Water leaching/min.–max. av.			0.01–77 6.7	0.1–60.0 13.4	0.5–13.4 4.4	1.2–5.4 3.3	1.9–12.1 6.9	0.7–4.0 2.2	0.01–3.8 0.7	0.4–11.8 4.1
0.01 M CaCl_2_ extraction/min.–max. av.			0.1–81.2 8.3	0.4–62.9 22.6	0.1–15.9 7.0	0.02–5.7 4.2	0.1–12.7 6.8	0.1–4.4 2.3	0.1–22.4 2.9	0.5–12.4 5.6
EDTA extraction/min.–max. av. (in mg/kg)			28–100 82 (1.20)	15–100 60 (0.04)	2–29 8 (0.07)	0.2–2 0.7 (0.02)	5–38 15 (0.22)	2–9 4 (0.14)	4–78 21 (0.47)	6–31 12 (1.45)
Plants^1^	(mg/kg)	Content in grass Toxic level Fodder plants for animals	0.23-0.33 >5 <50	0.05–0.6 – <0.5	0.03–0.12 15–50 0.05	0.6–3 5–30 3000	4–10 – 5–8	0.4–1.7 10–100 50–300	0.4–4.5 30–300 <30	12–72 100–400 <200
Animals^1^		Content in different land species	<1.5	0.1–1.5	<0.5	0.02–1.5	1–20	1–3	0.2–2	10–200

*min.* minimum, *max.* maximum, *av.* arithmetic average, “*–*” no data

^1^According Kabata-Pendias et al. (1999)

Of the metals considered, water leaching removes on the average 13% of the TC of Cd, slightly less—around 7% TCs of As and Cu—and lower quantities—around 4% TC of Co and Zn, 3% TC of Cr and around 2% TC of Ni. The lowest mobility in water extracts was determined for Pb, whose quantities ranged from 0.01 to 3.8% TC with an average of 0.7%.

Comparing the concentrations of the metals in the water extracts with the upper limits of metals in potable waters, the WHO (1993) criteria for Cr, Cu, Ni, Pb and Zn (0.05, 2.00, 0.02, 0.01 and 5 mg/dm^3^, respectively) are almost fully satisfied. Only does one result exceed the limit of 0.01 mg As per 1 dm^3^ As, and 16 samples do not meet the limit of 0.002 mg Co per 1 dm^3^.

The TCs of eluents extracted with 0.01 M CaCl_2_ from solid samples are in most cases higher than those water-leached (Table 4). The highest availabilities to living organisms reveal As and Cd: in the case of As in the range 0.1–81.2% TC with an average of 8.3% and in the case of Cd 0.4–62.9% TC with an average of 22.6%. Five elements have their eluent values significantly lower: below 23% TC of Pb (average 2.9%), below 16% TC of Co (average 7.0%), below 13% TC of Cu and Zn (average 6.8 and 5.6%, respectively) and considerably less of Ni below 4.4% TC (average 2.3%). The last metal—chromium—was extracted with 0.01 M CaCl_2_ in quantities from 0.02 to 5.7% TC.

The TCs of eluents extracted with 0.02 M EDTA from solid samples are in most cases considerably higher than those water-leached ones and higher than those extracted with 0.01 M CaCl_2_ (Table 4). The highest availabilities for plants reveal As and Cd: in the case of As in the range 28–100% TC with an average of 82% and in the case of Cd 15–100% TC with an average of 60%. Five elements have their eluent values significantly lower: below 78% TC of Pb (average 21%), below 38% TC of Cu (average 15%), below 31% TC of Zn (average 12%), below 29% TC of Co (average 8%) and considerably less of Ni—2–9% TC (average 4%). The quantities of the eighth metal—chromium extracted with 0.02 M EDTA (0.2–2% TC)—are lower than those leached with water (1.2–5.4% TC). It is a result of two possible forms of chromium that can occur in the environment. The Cr^6+^ compounds are soluble in water, mobile and, thus, harmful to plants and animals. The Cr^3+^ compounds are hardly soluble and, moreover, fast immobilized by proteins and stopped on cellular membranes (Kabata-Pendias and Mukherjee 2007a).

For comparative purposes, Table 4 presents contents of the elements considered in selected land-living organism, i.e. plants (mainly grass species) and animals, reported by Kabata-Pendias and Pendias (1999). The data are supplemented with the respective toxic data and permissible limits. The amounts of elements extracted with the 0.02 M solution of EDTA compared to the figures given of Kabata-Pendias and Pendias (1999) indicate the metals contained in the sands of the sandboxes not to be harmful to surrounding fauna and flora.

### Health risk of children

It is estimated that the daily intake of dirt by children via the digestive tract ranges between 39 and 270 mg, which is caused mainly by frequent putting dirty hands into their mouths, particularly when playing (Ljung et al. 2007). This behaviour may generate a specific exposure of children to poisoning with heavy metals and other chemical or microbiological components present among, on or within soil particles or sand grains. This threat is aggravated by a significantly higher index of metal absorption by children than by adults (US EPA 1986). Assuming 2 h daily that children spend in playgrounds and an accidental ingestion of sand grains of sandboxes, the authors calculated on the basis of the TC determinations the average daily doses [*D*_ing_, *D*_inhal_, *D*_dermal_, for calculation see Eqs. (3–5)] and the hazard quotients (HQ_ing,_ HQ_inhal,_ HQ_dermal_) of children 3 years old (accepting an average body mass of 15 kg) and 6 years old (accepting an average body mass of 20 kg). The reference doses (RfD) and slope factors (SF) were taken from IRIS (US EPA 2005) except those of Pb, for which the reference dose was calculated as a percent of the PTWI (permitted tolerable weekly intake) according to WHO (1993).

Besides the age groups, calculations of three HQ indexes (for non-carcinogens) and the risk index (for carcinogens) have been made distinguishing two groups of children: those permanently living in the spas (called residents) and those coming to the spas to spend usually 3-month-long recuperating or treating periods (called visitors). The children of both age groups of the residents and visitors alike are endangered mainly by metals entering their organisms due to the ingestion path: the HQ_ing_ indexes for all the elements considered are distinctly higher than the HQ_dermal_ and HQ_inhal_ indexes (Table 5). Cobalt is the single exception: its highest HQ_ing_ index is followed first by the HQ_inhal_ index and only then by the HQ_dermal_ one.

**Table 5:** Hazard quotient and cancer risk calculated for 3- and 6-year-old children (residents and visitors) for each element and exposure route in health resort

Element	RfD/SF^a^			Children									
				3-year-old resident^b^ visitors					6-year-old resident^b^ visitors				
	Ingestion	Inhalation	Dermal	HQ_ing_	HQ_inhal_	HQ_dermal_	ΣHQi	Risk	HQ_ing_	HQ_inhal_	HQ_dermal_	ΣHQi	Risk
	(mg/kg per day)												
As-non-cancer	3.00E−04		1.23E−04	*5.74E−02* 7.47E−03	*1.01E−06* 1.32E−07	*8.24E−03* 1.07E−03	*6.57E−02* 8.54E−03		*4.31E−02* 5.60E−03	*7.60E−07* 9.88E−08	*6.18E−03* 8.03E−04	*4.92E−02* 6.40E−03	
As-cancer	1.50E+00	1.51E+01	3.66E+00	*1.11E−06* 1.44E−07	*1.95E−10* 2.54E−11	*1.59E−07* 2.07E−08		*1.27E−06* 1.65E−07	*1.66E−06* 2.16E−07	*2.93E−10* 3.81E−11	*2.38E−07* 3.10E−08		*1.90E−06* 2.47E−07
Cd-non-cancer	1.00E−03		1.00E−05	*7.08E−04* 9.21E−05	*1.25E−08* 1.62E−09	*1.39E−04* 1.80E−05	*8.47E−04* 1.10E−04		*5.31E−04* 6.90E−05	*9.37E−09* 1.22E−09	*1.04E−04* 1.35E−05	*6.35E−04* 8.26E−05	
Cd-cancer		6.30E+00			*3.37E−12* 4.39E−13			*3.37E−12* 4.39E−13		*5.06E−12* 6.58E−13			*5.06E−12* 6.58E−13
Co-non-cancer	2.00E−02	5.71E−06	1.60E−02	*5.13E−04* 6.67E−05	*3.17E−05* 4.13E−06	*1.26E−06* 1.64E−07	*5.46E−04* 7.10E−05		*3.85E−04* 5.01E−05	*2.38E−05* 3.09E−06	*9.43E−07* 1.23E−07	*4.10E−04* 5.33E−05	
Co-cancer		9.80E+00			*7.61E−11* 9.89E−12			*7.61E−11* 9.89E−12		*1.14E−10* 1.48E−11			*1.14E−10* 1.48E−11
Cr-non-cancer	3.00E−03	2.86E−05	6.00E−05	*1.71E−02* 2.22E−03	*3.16E−05* 4.11E−06	*1.67E−03* 2.18E−04	*1.88E−02* 2.44E−03		*1.28E−02* 1.66E−03	*2.37E−05* 3.08E−06	*1.25E−03* 1.63E−04	*1.41E−02* 1.83E−03	
Cr-cancer		4.20E+01			*1.63E−09* 2.12E−10			*1.63E−09* 2.12E−10		*2.44E−09* 3.17E−10			*2.44E−09* 3.17E−10
Cu	4.00E−02		1.20E−02	*4.54E−04* 5.91E−05	*8.02E−09* 1.04E−09	*2.97E−06* 3.86E−07	*4.57E−04* 5.95E−05		*3.41E−04* 4.43E−05	*6.01E−09* 7.82E−10	*2.23E−06* 2.89E−07	*3.43E−04* 4.46E−05	
Ni-non-cancer	2.00E−02		5.40E−03	*2.03E−03* 2.64E−04	*3.58E−08* 4.66E−09	*1.47E−05* 1.92E−06	*2.04E−03* 2.66E−04		*1.52E−03* 1.98E−04	*2.69E−08* 3.49E−09	*1.10E−05* 1.44E−06	*1.53E−03* 1.99E−04	
Ni-cancer		8.40E−01			*2.58E−11* 3.35E−12			*2.58E−11* 3.35E−12		*3.87E−11* 5.03E−12			*3.87E−11* 5.03E−12
Pb	3.50E−03		5.25E−04	*6.91E−03* 8.99E−04	*1.22E−07* 1.59E−08	*9.03E−05* 1.17E−05	*7.00E−03* 9.10E−04		*5.18E−03* 6.74E−04	*9.15E−08* 1.19E−08	*6.77E−05* 8.81E−06	*5.25E−03* 6.83E−04	
Zn	3.00E−01		6.00E−02	*5.31E−04* 6.90E−05	*9.37E−09* 1.22E−09	*5.20E−06* 6.77E−07	*5.36E−04* 6.97E−05		*3.98E−04* 5.18E−05	*7.03E−09* 9.14E−10	*3.90E−06* 5.07E−07	*4.02E−04* 5.23E−05	

^a^RfD (reference dose) and SF (slope factor) for all elements according to US EPA IRIS (2005) and for Pb according to WHO (1993)

^b^Italics: for residents accepted EF = 646 h/year, for visitors EF = 84 h/year

The highest HQ_ing_ indexes are those of As and Cr. For both age groups (3 and 6 years olds), they are 5.74E−02 and 1.71E−02, respectively, for residents, while 4.31E−02 and 1.28E−02, respectively, for visitors. The difference (larger figures) for the elder children results simply from their higher body mass. The values of the HQ_ing_ indexes of the remaining metals are lower than those of As and Cr; thus, the decreasing sequence of the elements is As >Cr > Pb > Ni > Cd > Zn > Co > Cu. Within the group of the visitors, the HQ_ing_ is distinctly lower and ranges from 7.47E−03 of As to 5.91E−05 of Cu for the children 3 years old, while from 5.60E−03 of As to 4.43E−05 of Cu for the children 6 years old.

Considering the dermal path of children exposure, the relatively highest are the HQ_dermal_ indexes of As: 8.24E−03 and 6.18E−03 for the 3 and 6 years olds, respectively. In the case of the remaining elements, they decrease in the sequence As > Cr > Cd > Pb > Ni > Zn > Cu > Co, which differs from the sequence established for HQ_dermal_. Comparing the HQ_dermal_ values of the residents and the visitors, the figures for the visitors are about one order of magnitude lower due obviously to a shorter exposure.

The contribution of inhaling is almost of the lowest health risk for almost all the metals considered. The highest HQ_inahl_ indexes are in the case of Co: being 3.17E−05 and 4.13E−06 for the 3-year-old residents and the visitors, respectively, and 2.38E−05 and 3.09E−06 for the 6-year-old residents and visitors, respectively. The HQ_inahl_ indexes of Cr are almost the same as those of Co. The values of indexes of the remaining metals are lower; thus, the sequence is Co = Cr > As > Pb > Ni > Cd > Zn > Cu.

The total hazard index (*HI*) for the 3 years olds is 9.59E−02 (residents) and 1.25E−02 (visitors), while for the 6 years olds is 7.19E−02 (residents) and 9.35E−03 (visitors).

In the case of carcinogens, the highest risk has been calculated for As. The risk values of the 3 years olds are 1.27E−06 and 1.65E−07 (residents and visitors, respectively) while those of the 6 years olds 1.90E−06 and 2.47E−07 (residents and visitors, respectively). The risk values of the elements considered decrease for both age groups in the sequence As > Cr > Co > Ni > Cd.

With regard to the upper limits set for the two indexes, the total health risk (*HI*) values in the case of all the elements considered are below a figure of 1, while in the case of the cancerogenic substances are below a figure of 1.10E−05. Such results prove that the children playing in the sandboxes of the seven spas of Poland are not at endangered by the metals that the sandboxes contain.

## Discussion and conclusions

Making a comparison of current data to those of other authors is difficult as the results depend mainly on different parameters accepted in calculating the HQ, such as the body mass, daily intake of soil (dirt) ingested and number of days or hours spent in playgrounds. Other reasons include sample types, sampling methods, contributions of the fractions separated to the total sample and extraction methods of metals from the solid sample. Therefore, the authors have decided to compare only the totals of metals in sandy materials or soils.

The sands of the sandboxes of Cracow, which is an agglomeration of almost a million of inhabitants located approximately up to 100 km south of the spas considered (except Swoszowice), i.e. immediately outside the Carpathians, were studied by Jasiewicz et al. (2009). The mean contents of Cd, Pb and Zn of the spas (Table 6) are significantly lower: 0.06, 2.05 and 13.5 mg/kg, respectively, than those of Cracow: 0.08, 13.7 and 66.24 mg/kg, respectively. Comparable are the means of Cu: 1.27 (range 0.56–2.24 mg/kg) of the spas and 1.54 (range 0.65–4.95 mg/kg) of Cracow. The means of Cr and Ni of the spas are around two times higher: 4.34 and 3.44 mg/kg, respectively, than those of Cracow: 2.28 and 1.72 mg/kg, respectively.

**Table 6:** Comparison of the result of this study with those of other contents of metals in sand and soil studies

Material	Site	Methods	As		Cd		Co		Cr		Cu		Ni		Pb		Zn	
			Range	Av.	Range	Av.	Range	Av.	Range	Av.	Range	Av.	Range	Av.	Range	Av.	Range	Av.
			(mg/kg)															
Sand	Kraków Poland^a^	HNO_3_ + HClO_3_ 1 M HCl	−	−	0.00–0.30 0.03–0.48	0.08 0.14	−	−	1.0–13.75 0.04–0.52	2.28 0.16	0.65–4.95 0.03–2.94	1.27 0.79	0.73–6.98 0.00–1.41	1.72 0.22	1.83–46.75 0.18–33.40	13.7 8.8	7.10–210 0.52–147.0	66.24 39.89
	Bytom Katowice Sosnowiec Bukowno, Poland^b^	−	−	−	<0.11–0.69	− 0.18 3.22 2.37	−	−	−	−	−	−	−	−	<0.46–19.24	− 115.4 96.9 77.6	6.12–81.35	− 246.00 521.97 363.87
	Silesia, Poland^c^	HNO_3_ + HCl	0.1–0.65	0.28	1–3	2	5–14	9.8	−	−	0.4–4	1.1	4–9	6.8	25–40	31	7–285	57
	this study	HNO_3_ + HCl	0.2–3.6	1.46	0.01–0.18	0.06	0.21–1.59	0.87	2.40–6.02	4.34	0.56–2.24	1.54	1.46–5.37	3.44	0.68–5.50	2.05	7.59–37.92	13.50
Sandy substrate	Madrid, Spain^d^	HNO_3_ + HCl	3.7–14	6.9	0.05–0.3	0.14	1.5–5.8	3.2	4.1–51	17	5.4–39	14	2–14	5.7	6.1–65	22	20–103	50
Soil	Silesia Poland^c^	HNO_3_ + HCl	2.3–6.1	3.7	3–12	7.4	7–19	11	−	−	5–45	31	7–40	16.5	65–789	247	27–365	166
	Poznan Poland^e^	6 M HCl	−	−	0.22–7.16	−	−	−	−	−	1.1–124.5	−	−	−	2.5–95.9	−	6.1–225.5	−
	Owerri, Nigeria^f^	4 N HNO_3_	−	−	0.31–1.45	0.76	0.69–13.21	4.76	3.9–27.2	12.80	11.6–76.8	26.56	2.2–61.53	20.31	4.55–7.19	6.0	32.34–168.7	62.34
	Istanbul, Turkey^g^	HNO_3_ + HCl + HF	−	−	1.21–2.07	−	0.18–36.87	16.94	1.1–102	61.68	3.18–57.7	25.79	1.12–59.5	31.48	18.26–73.20	33.9	81.6–242.4	154.24
	Sao Paulo Brazil^h^	INAA	1.3–24	−	−	−	−	−	21–70	−	−	−	−	−	−	−	15–179	−

*av.* average, “*–*” no data

^a^Jasiewicz et al. (2009)

^b^Nieć et al. (2013)

^c^Kicińska (2016a, 2016b)

^d^De Miguel et al. (2007); data for 2003 year

^e^Diatta and Grzebisz (2011)

^f^Verla et al. (2015)

^g^Namik et al. (2012)

^h^Figueiredo et al. (2011), *INNA* Instrumental Neutron Activation Analysis

Considerable differences in the mean Cd, Pb and Zn contents of sandbox sands occur between the spas (Table 6) and the region of Silesia (Kicińska 2016a, b; Nieć et al. 2013), the latter being the most industrialized region of Poland. They are even “several tens times lower than those of the Silesian playgrounds, where Cd ranges from 0.18 to 3.22, Pb from 31 to 115 and Zn from 57 to 522 mg/kg (the values refer to the towns Katowice and Sosnowiec, respectively). Comparable results were established by De Miguel et al. (2007) for Madrid: the mean contents of As, Cd, Co, Cr, Ni and Zn in Madrid were two to five times higher whereas of Pb and Cu around 9–10 lower than those in the spas of S Poland.

Considering soils of playgrounds, their significantly high contents of metals and similar distribution trends as in Cracow and Silesia (op. cit.) were noted in Owerri in Nigeria (Verla et al. 2015), Istanbul in Turkey (Namik et al. 2012) and Sao Paulo in Brazil (Figueiredo et al. 2011). However, these are all urban, not spa-status localities.

Although the *HI* and risk indexes are below the values of a possible health threat for children, the authors recorded a significant pollution of the environment with metals, among which As and Cr are the major contributors. Both enter the human organism due to skin penetration and via the digestive tract (inadvertent swallowing of dirt); their entering via lungs when breathing is of minor importance.

Compounds of arsenic show affinity to many enzymes. They accumulate in the bones, liver and kidneys and also in the tissues rich in keratin (Kabata-Pendias et al. 1993). A long-lasting exposure to As and its excessive amounts in humans result in skin alterations and disorders in the functioning of the blood circulation, nervous and breathing systems. Arsenic is also a strong carcinogenic element (Zwozdziak et al. 2016).

Chromium occurs in the environment due to its chemical properties and relatively high resistance of Cr^3+^ compounds to weathering. Therefore, Cr^3+^ often accumulates in the hardly soluble residuum but, under oxidizing conditions, is adsorbed as Cr^6+^ by clay minerals and oxy/hydroxides of Fe and Al (Kicińska 2011). In the human organism, Cr may accumulate in the brain, spinal cord and kidneys. Together with As, chromium accumulates in children’s hair and milk teeth, in which the two metals may be indicative of an overall metal pollution of the environment (Kicińska and Jelonek-Waliszewska 2017). Long-lasting exposure to Cr disturbs the circulatory and respiratory systems and results in skin diseases, while the chromic acid may seriously injure internal organs.

Chromium Cr^6+^ and ions of the remaining metals considered are bound to clay minerals as well as to Fe and Mn oxy/hydroxides; these mineral phases form the finest grain fraction of the sands sampled in the current study. The grains of quartz that is chemically an inert mineral dominate in the samples. Therefore, the HQ index in the case of such quartz-rich materials as sands should be calculated on the basis of their finest fractions. A separate paper dealing with this issue is under preparation.

The problem of children exposure to arsenic in sandy substrate soils of Madrid playgrounds was dealt with by De Miguel et al. (2007), who determined 3.7–14 mg/kg As. The present authors have established in the sands of the Polish spas 0.2–3.6 mg/kg As and identified this element to be the largest single contributor to the overall health risk of children. The *HI* indexes of As, Pb, Cd and Cu: 1.09E−01, 3.11E−02, 1.09E−03 and 1.41E−03, respectively, calculated by De Miguel et al. (2007) for the children 6 years old in Madrid are considerably lower than those measured here in the Polish spas: 4.92E−02, 5.25E−03, 6.35E−04 and 3.43E−04, respectively. The *HI* index of Ni in the sands of the Polish spas is slightly higher than that of Madrid (1.53E−03 and 9.6E−04, respectively), whereas the *HI* indexes of Zn and Cr are almost comparable (7.10E−04 versus 4.02E−04, and 2.72E−02 versus 1.41E−02, respectively). The risk indexes calculated for cancerogens of the Polish spas are comparable only in the case of As to those of the Madrid parks, while significantly lower (at least by one order of magnitude) in the case of Cd, Ni and Cr than the figures for Madrid.

Summarizing, the following conclusions can be drawn for the investigations of seven Polish spas:

1. The total values of the metals considered, i.e. As, Cd, Co, Cr, Cu, Ni, Pb and Zn, determined in the sands of the spa sandboxes are below the values permissible for the soils of the protected areas (type A).
2. The mobility of these metals is low and their fractions extracted with water contribute to the total metal contents between 0.7% TC of Pb and 13% TC of Cd.
3. The phytoavailable metal fractions extracted with EDTA and CaCl_2_ contribute in general considerably more to the total metal contents than do the water-leached fractions. The contribution of As is as high as 82% TC on the average, however that of Cr only 0.7% TC (in EDTA-extraction). In the case of CaCl_2_ extraction, the values are much lower and range from slightly above 2% TC of Ni to the highest 23% TC of Cd. The values obtained do not represent a significant threat to the soil-plant environment.
4. The total health risk indexes *HI* calculated for non-cancerogenic substances amount to 1.25E−02 and 9.35E−03 for the children 3 and 6 years old, respectively, who are permanent spa residents. For the children who are only spa visitors, the *HI* indexes are much lower and amount to 9.58E−02 and 7.19E−02, respectively. The incidentally ingested arsenic is the major contributor to the indices, and its values for the children residing in spas are 5.74E−02 for the 3 years olds and 4.31E−02 for the 6 years olds. However, in the case of all the elements analysed, their *HI* values are significantly below a level of 1 and prove the lack of hazardous impacts on children.
5. The highest risk values in the case of cancerogenic substances are those of As: 1.27E−06 and 1.90E−06 for 3- and 6-year-old residents, respectively, and 1.65E−07 and 2.47E−07 for 3- and 6-year-old visitors. Considerably lower values have been obtained for Cr > Co > Ni > Cd. As all these figures as below the permissible limit (1.10E−05), thus the risk factors of the metals considered are insignificant.
6. Despite a lack of the low health risk, an adverse overall impact of road traffic (the presence of such metals as Cu, Zn, Ni, Cr, Co and Pb) and of low emissions (mainly As and Cd) on the state of the environment in the spas has been established.
